# 乙二胺二琥珀酸功能化硅胶协同去除孔雀石绿及Cr(Ⅵ)

**DOI:** 10.3724/SP.J.1123.2023.12008

**Published:** 2024-10-08

**Authors:** Lu YAO, Min HE, Hongbin HU, Lang ZHAO, Yuwei LÜ, Rong LI

**Affiliations:** 西北大学化工学院, 陕西 西安 710069; School of Chemical Engineering, Northwest University, Xi’an 710069, China

**Keywords:** 乙二胺二琥珀酸, 孔雀石绿, 铬(Ⅵ), 非均相类Fenton催化, 降解和去除, 密度泛函理论, 分子轨道, ethylenediamine disuccinic acid (EDDS), malachite green (MG), Cr(Ⅵ), heterogeneous fenton-like catalysis, degradation and removal, density functional theory (DFT), molecular orbital

## Abstract

孔雀石绿(MG)与Cr(Ⅵ)普遍共存于印刷、皮革和纺织等工业领域的废液中,给人类及生态环境造成严重危害。开发从废水环境中协同去除MG及Cr(Ⅵ)的有效方法具有重要的研究价值。该工作以乙二胺二琥珀酸(EDDS)及硅胶(Silica)为原料,成功制备了非均相类芬顿(Fenton)催化剂EDDS-Silica、EDDS-Co^2+^-Silica,并进一步开发了一种非均相类Fenton催化法高效协同去除废水中MG及Cr(Ⅵ)的新工艺。在MG一元体系的降解过程中,两种材料的加入均可克服传统Fenton反应仅适用于酸性环境的限制,且EDDS-Co^2+^-Silica对MG表现出更优越的降解效果。通过密度泛函理论和分子轨道计算,预测了金属Co^2+^与EDDS-Silica的最佳配位方式,以及MG在降解过程中易捕获或逸出电子的位点。二元体系降解实验发现,在不同影响因素下,EDDS-Co^2+^-Silica对Fenton反应仍具有显著促进效果,且MG与Cr(Ⅵ)之间存在正向协同作用。在此基础上,利用EDDS配体优越的金属螯合特性,将EDDS-Silica作为吸附剂用于去除Fenton反应后残余的、不同价态的总Fe和总Cr。结果表明,本研究提出的氨基多羧酸类改性材料作为非均相类Fenton催化剂,具备促Fenton反应和去除残留金属离子的双重性质,在有效处理MG及Cr(Ⅵ)共存废水的同时,确保体系中残留金属离子含量均满足环境排放标准。该研究在染料降解和重金属离子废水处理领域具有广泛应用前景,为类似配体改性材料的开发提供了参考价值和理论依据。

## Abstract (en)

As common industrial raw materials, malachite green (MG) and Cr(Ⅵ) generally coexist in waste liquids discharged from the paper printing, leather, and textile industries, causing serious harm to humans and the environment. Therefore, developing an effective method for the synergistic removal of MG and Cr(Ⅵ) from aquatic environments is of great research value.

In this work, the non-homogeneous Fenton-like catalysts, namely, EDDS-Silica and EDDS-Co^2+^-Silica were successfully prepared using ethylenediamine disuccinic acid (EDDS) and silica gel (Silica) as raw materials, and a non-homogeneous Fenton-like catalytic method was developed for the efficient and synergistic removal of MG and Cr(Ⅵ) from wastewater. EDDS-Silica and EDDS-Co^2+^-Silica were analyzed using Fourier infrared spectroscopy and X-ray photoelectron spectroscopy to determine their structural composition and elemental contents. The catalytic degradation and removal effects of these materials in an MG single-waste system were also investigated. The results demonstrated that the incorporation of both materials can overcome the limitation of the conventional Fenton reaction, which is its applicability to acidic environments only. Moreover, EDDS-Co^2+^-Silica showed better degradation effects on MG than EDDS-Silica. Quantitative calculations based on density functional theory were used to predict the optimal coordination forms between Co^2+^and EDDS-Silica as well as the MG structure. The lowest unoccupied and highest occupied molecular orbitals of the catalysts were then used to predict the active sites on which MG tends to capture or release electrons during the degradation reaction. The optimal conditions for the synergistic removal of MG and Cr(Ⅵ) from a binary system using EDDS-Co^2+^-Silica were further investigated under different influencing factors. The results showed that EDDS-Co^2+^-Silica still had excellent catalytic effect on the degradation rate of MG in the range of pH 3-7, and the optimal conditions were as follows: solution pH, 7; degradation time, 1 h; temperature, 25 ℃; H_2_O_2_ concentration, 20 mmol/L; and the initial mass concentration of Cr(Ⅵ), 25 mg/L. Under the above conditions, the degradation rate was increased from 87.25% to 96.67% compared with that in the MG monosystem. Obvious enhancements in degradation effect and efficiency confirmed that the incorporation of EDDS-Co^2+^-Silica was favorable for the synergistic removal of MG and Cr(Ⅵ) in the binary system. Strongly oxidizing Cr(Ⅵ) can participate in the Fenton reaction, thus promoting MG degradation over a wide pH range. Thus, a positive synergistic effect exists between MG and Cr(Ⅵ). Considering that a large number of metal ions remained in the solution after the degradation reaction, EDDS-Silica was added to the degradation solution, and adsorption experiments were performed for 4 h at 30 ℃to adsorb and remove Cr and Fe via the strong chelating property of EDDS. The total residual mass concentrations of Cr and Fe were 4.96 and 1.02 mg/L, respectively, which meet national emission standards. These findings indicate that EDDS-Silica has good effects on the removal of residual metal ions after the nonhomogeneous Fenton reaction. As heterogeneous Fenton-like catalysts, the aminopolycarboxylic acid-modified materials proposed in this study can simultaneously promote the Fenton reaction and remove residual metal ions, thereby effectively removing MG and Cr(Ⅵ) from the binary system while ensuring that the content of residual metal ions in the system meets environmental emission standards. This study has broad application prospects in dye degradation and heavy-metal-ion wastewater treatment, and provides a reference value and theoretical basis for the development of other similar ligand-modified materials.

随着工业化快速发展,染料及重金属导致的水污染成为全球性问题。纺织、印刷、皮革等行业每年消耗的染料高达10000 t,其中10%~15%的染料废水未经处理排入水介质^[1]^,造成环境污染、生态失衡。孔雀石绿(MG)是一种阳离子三苯基甲烷碱性染料^[2]^,广泛应用于纺织业、印染工业等领域^[3]^。MG是潜在的致畸、致癌物质,不仅会抑制生物体的生长,影响其生育率^[4]^,还会对人类的皮肤、肾脏和心脏及骨骼造成损害^[5]^。与此同时,在皮革、印染工业等领域的制造过程中,还会引入有毒物质,如重金属六价铬Cr(Ⅵ)。Cr(Ⅵ)作为固定染料的常用媒染剂,通常在实际废水中与MG共存^[6,7]^。Cr(Ⅵ)会间接导致肾炎和胃肠道溃疡的发生,而且是致癌物^[8]^。Cr(Ⅵ)即使从污水中分离出来,也会在生物链内再次迁移,由此造成环境的二次污染^[9]^。由此可见,将高毒性的Cr(Ⅵ)转化为低毒的Cr(Ⅲ)具有很高的环境价值。因此,开发从水生环境中协同去除MG和Cr(Ⅵ)的技术是一个重要的研究领域。

常见废水中去除MG及Cr(Ⅵ)的方法有吸附法^[10⇓-12]^,微生物法^[13]^,光、电催化法^[14,15]^。在众多方法中,吸附法设备及操作简单,但只是将污染物从液相转移到固相,会产生二次污染;微生物法涉及的水环境复杂,降解条件不易控制;光、电催化法虽引入光照条件或电极催化从而提高降解效率,但苛刻的反应条件、额外的能耗以及催化剂难回收等问题,均会导致成本过高而不利于实际推广。

高级氧化工艺(AOPs)是城市水和废水三级处理的一种有效解决方案^[16]^。Fenton催化技术是AOPs中最为广泛使用的方法之一^[17⇓-19]^,然而,传统Fenton法仅适用于pH<3的强酸性废液^[20]^,且反应后会产生大量的铁污泥,造成大量铁污泥悬浮物在废水中难以降解,会影响水生生物的繁殖,破坏生态系统的平衡^[21]^,这使该法的推广和应用均受到限制。为了克服上述不足,Hu等^[22]^在Fenton体系中加入液态螯合剂乙二胺四乙酸(EDTA),可在均相中与铁离子形成稳定的配合物,阻止铁污泥的生成。但该配体具有低生物降解性,会在均相反应体系中产生二次污染。此外,均相体系中添加其他过渡金属元素亦可提高Fenton反应的降解效率,例如Co^2+^的加入可以提高整个体系的催化活性^[23]^。然而,均相体系中Co^2+^的加入一方面会对环境造成危害,另一方面存在着催化剂回收、分离和再生等一系列问题,这在很大程度上限制了钴基材料作为类Fenton催化剂的应用。

作为一个绿色环保并可提供6个配位原子的螯合配体,乙二胺二琥珀酸(EDDS)被认为是EDTA的合适替代品^[24]^,利用六齿配体EDDS优越的金属螯合特性可与Co^2+^形成稳定的配合物,进而作为类Fenton催化剂参与非均相类Fenton反应。为了克服上述均相Fenton反应的局限性,本研究采用EDDS和EDDS-Co^2+^对硅胶材料进行改性制得绿色环保、便于回收的类Fenton催化剂固体材料EDDS-Silica和EDDS-Co^2+^-Silica。在非均相Fenton体系下,EDDS-Silica和EDDS-Co^2+^-Silica作为类Fenton催化剂以期有效协同去除模拟废液中的MG和Cr(Ⅵ),并确保废液中残留的总Fe和总Cr满足排放标准。

## 1 实验部分

### 1.1 仪器与试剂

ULTIMA 2X电感耦合等离子体发射光谱仪(ICP-AES,法国HORIBA Jobin Yvon公司); EQUINOX-55傅里叶红外光谱仪(FTIR,德国Bruke公司)、Nexsa X射线光电子能谱(XPS,美国Thermo Fisher公司); STA449F3热重分析仪(德国Netzsch公司); JH721紫外分光光度计(上海菁华科技仪器有限公司); SHA-82A数显水浴恒温振荡器(常州普天仪器制造有限公司); HWCL-3恒温磁力搅拌浴(郑州长工贸有限公司);UPD-II-10T纯水仪(西安优普仪器设备有限公司); ZDJ-4B电位滴定仪、PHS-25 pH电位仪(上海仪电科学仪器股份有限公司)。

硅胶(8 μm,苏州纳微科技股份有限公司); EDDS(纯度98%,上海麦克林公司); 3-缩水甘油基氧基丙基三甲氧基硅烷(*γ*-GLDP、纯度95%,辽宁盖县化工研究所); MG (纯度95%,天津大茂化学试剂厂);六水合氯化钴(CoCl_2_·6H_2_O)、七水合硫酸亚铁(FeSO_4_·7H_2_O)、重铬酸钾(K_2_Cr_2_O_7_)、盐酸(HCl)、氢氧化钠(NaOH)、过氧化氢(H_2_O_2_(30%))等分析级试剂,均购自科密欧化学试剂有限公司。

### 1.2 MG及Cr(Ⅵ)标准溶液配制及标准曲线制作

准确称取0.2829 g K_2_Cr_2_O_7_和0.1000 g MG,分别用超纯水溶解并定容至100 mL容量瓶中,得到质量浓度为1000 mg/L的MG及Cr(Ⅵ)标准储备液。准确吸取MG标准储备液25.00 mL于500 mL容量瓶中,用纯水稀释得到质量浓度为50 mg/L的MG一元废水模拟液。准确吸取25.00 mL MG和12.50 mL Cr(Ⅵ)标准储备液,混合至500 mL容量瓶内,用纯水稀释得到含MG和Cr(Ⅵ)的二元废水模拟液,其中MG及Cr(Ⅵ)的质量浓度分别为50 mg/L和25 mg/L,后续采用HCl或NaOH调节至所需pH,得到标准工作溶液。

配制质量浓度为50 mg/L的MG水溶液,分别取0.25、0.5、1.0、2.0、3.0、4.0、5.0 mL置于7支25 mL的比色管中,并加入超纯水定容至刻度,反复摇匀,即得0.5~10 mg/L的孔雀石绿标准溶液。用纯水作为对比,用紫外可见分光光度计在616 nm波长下进行吸光度测试,制作标准曲线,线性方程为*A*=0.0506*C*-0.0204, *r*^2^=0.9992(*A*为吸光度,*C*为孔雀石绿溶液的质量浓度,*r*^2^为相关系数)。后续降解实验中孔雀石绿的含量均由该方程计算得出。

采用GB 7467-87二苯碳酰二肼显色分光光度法用紫可见分光光度计在540 nm波长下进行吸光度测试,制作标准曲线,线性方程为*A*=1.1614*C*+0.0081, *r*^2^=0.9996。后续降解实验中Cr(Ⅵ)的含量均由该方程计算得出。

### 1.3 材料的制备

参考武晓梅等^[25]^方法,改性修饰Silica材料。将6 g EDDS置于80 mL H_2_O中,调节pH为11.0,转移至烧瓶后逐滴滴入4 mL *γ*-GLDP,分散均匀静置1 h后于65 ℃下反应12 h。反应停止后调节溶液pH至4。将1.5 g硅胶添加至反应溶液中,在90~95 ℃下搅拌反应2 h,用10%乙酸冲洗3次后用超纯水洗涤反应产物至中性,于65 ℃下真空干燥12 h,得到EDDS-Silica改性材料(见图1)。

**图1:**
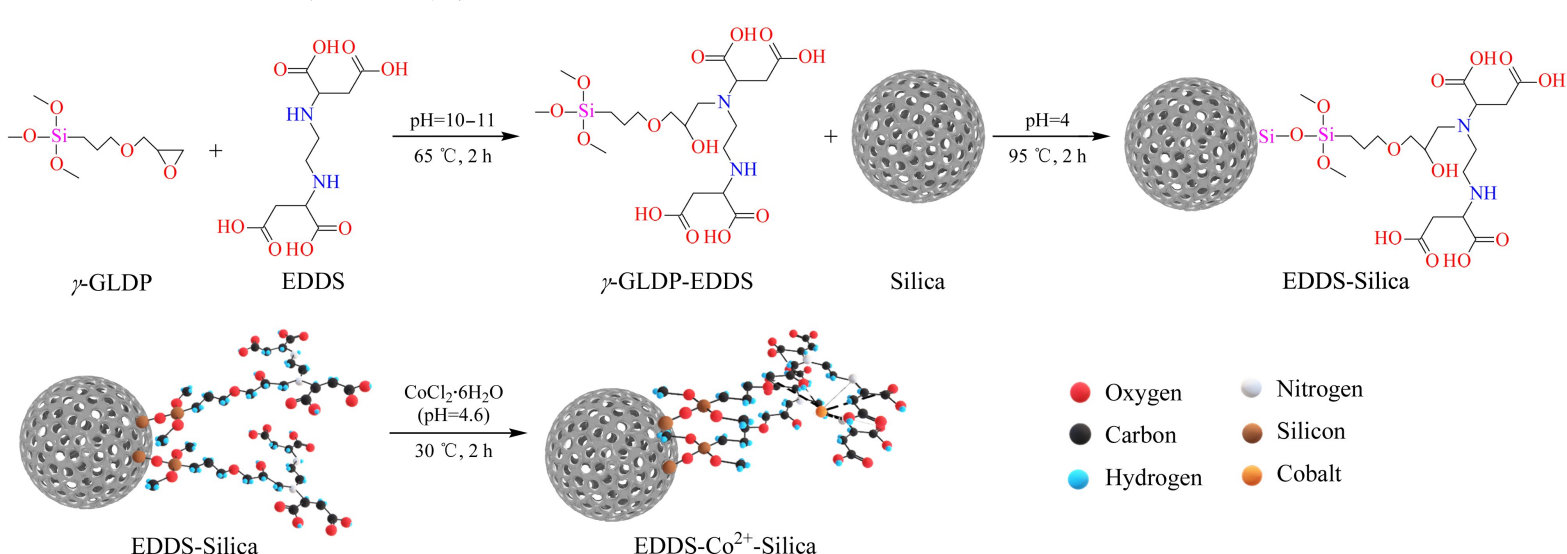
EDDS-Silica和EDDS-Co^2+^-Silica的制备流程图

准确称取1.0 g(精确至0.0001 g)EDDS-Silica,置于100 mL锥形瓶中,加入20 mL CoCl_2_·6H_2_O溶液(1 mmol/L、pH 4.6),超声混合均匀,密封,置于恒温水浴振荡器中,在30 ℃、200 r/min条件下吸附2 h至平衡,收集反应产物,于65 ℃下真空干燥12 h,制得EDDS-Co^2+^-Silica材料(见图1)。

### 1.4 表征

分别对EDDS-Silica、EDDS-Co^2+^-Silica材料进行FTIR、XPS表征分析,确定材料结构组成和元素含量。参照文献[26],采用电位滴定法(TA),确定改性材料上EDDS的键合量。采用ICP-AES测定吸附前后溶液中Co^2+^的含量,按照式(1)计算Co^2+^键合量。


(1) $$q_{e}=\frac{\left(C_{0}-C_{e}\right)V}{m}$$

其中,*q*_e_: Co^2+^的平衡键合量,mg/g; *C*_0_: Co^2+^的初始含量,mg/L; *C*_e_: Co^2+^的平衡含量,mg/L; *V*:溶液体积,L; *m*: EDDS-Silica材料质量,g。

### 1.5 密度泛函理论(DFT)计算

采用Gaussian 16软件,以B3LYP和6-31G(d)为基组分别对金属Co与EDDS的螯合结构以及MG结构进行优化。按照式(2)计算优化前后结构的能量差。推测EDDS-Co^2+^-Silica材料中金属Co与EDDS的配位方式及MG在降解过程中的最佳活性位点。


(2) Δ*E*=*E*_EDDS-Co_-(*E*_EDDS_+*E*_Co_)

式中,Δ*E*为结构优化前后的能量差,*E*_EDDS-Co_为EDDS-Co配位结构优化后的能量,*E*_EDDS_和*E*_Co_分别为EDDS和Co结构优化前的能量,单位均为eV。

### 1.6 催化性能测试

#### 1.6.1 MG一元体系的降解实验

称取0.0030 g EDDS-Silica、0.0030 g EDDS-Co^2+^-Silica,分别加入50 mL MG模拟液中,同时加入Fenton试剂FeSO_4_·7H_2_O(终浓度100 μmol/L)、H_2_O_2_(终浓度20 mmol/L)。密封后置于恒温水浴振荡器中,在25 ℃、200 r/min条件下进行降解反应1 h后采用针式过滤器过滤。以不添加材料的Fenton反应为对照实验,在616 nm波长下测定MG的吸光度,按照式(3)计算MG的降解率(*R*)。


(3) $$R=\frac{C_{1}-C_{t}}{C_{1}}\times 100\%$$

式中,*C*_1_为待测物初始含量,mg/L; *C_t_*为待测物平衡含量,mg/L。

#### 1.6.2 MG及Cr(Ⅵ)二元体系的降解实验

准确称取0.0030 g EDDS-Co^2+^-Silica材料加入50 mL 二元废水模拟液(pH 7)中,同时加入Fenton试剂FeSO_4_·7H_2_O(终浓度100 μmol/L)、H_2_O_2_(终浓度20 mmol/L),密封后于25 ℃恒温水浴振荡器中降解1 h(200 r/min)。探究不同影响因素对二元废水模拟液协同去除情况的影响。在540 nm波长下测定Cr(Ⅵ)的吸光度;按照式(3)分别计算MG的降解率和Cr(Ⅵ)的去除率。

### 1.7 吸附性能测试

取1.6.2节降解反应后残余液20 mL(pH 7),加入0.3000 g EDDS-Silica,密封后于30 ℃恒温水浴振荡器中吸附4 h(200 r/min),取上清液,采用ICP-AES测定处理前后模拟液中总Cr及总Fe浓度,探究不同影响因素对EDDS-Silica吸附性能的影响,按照式(3)计算总Cr去除率。

## 2 结果及讨论

### 2.1 改性材料的表征分析

Silica、EDDS-Silica和EDDS-Co^2+^-Silica的FT-IR表征结果如图2所示。与Silica的谱图对比,EDDS-Silica及EDDS-Co^2+^-Silica均在2885、2823、1733、1400、1218、1095、808 cm^-1^处出现新的吸收峰,分别对应CH_3_、CH_2_、C=O、C-H、C-N、Si-O-Si、Si-C键的伸缩振动;将波长范围筛选到500~900 cm^-1^,与EDDS-Silica的谱图对比,EDDS-Co^2+^-Silica在521~774 cm^-1^的范围内出现新的吸收峰,对应Co-O和Co-N键的伸缩振动^[27]^。该结果表明,金属Co^2+^与EDDS-Silica发生相互作用,成功键合到EDDS-Silica上。

**图2:**
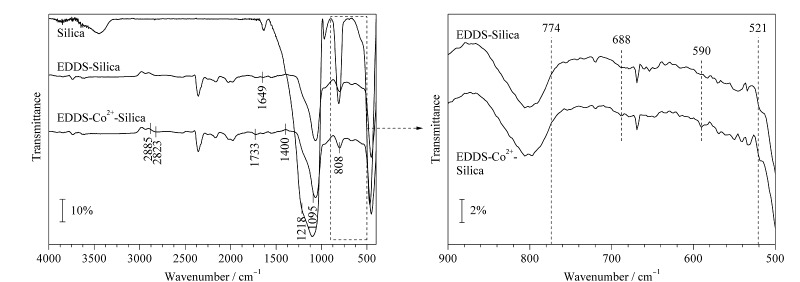
改性材料的傅里叶红外光谱图

由表1和图3a可知,与Silica材料相比,EDDS-Silica新增N元素峰,且相对含量由0增至2.54%, C元素峰增大,O、Si元素峰有所减小;与EDDS-Silica相比,EDDS-Co^2+^-Silica新增Co元素峰,相对含量增至0.33%,其他元素峰的含量也相对调整。由EDDS-Silica的C 1*s*分峰拟合谱图可知(图3b), 5个主峰284.23、284.78、285.38、286.48和289.23 eV分别对应C-Si、C-C、C-O-C、C-N和O=C-O官能团;EDDS-Silica的Si 2*p*分峰拟合谱图表明(图3c), 2个主峰102.02、103.72 eV分别对应C-Si-O和Si-O-Si官能团。上述结果证明成功制得EDDS-Silica、EDDS-Co^2+^-Silica材料。

**表1:** 改性材料的相对原子含量

Sample	C/%	O/%	Si/%	N/%	Co/%
Silica	15.37	55.14	29.49	0.00	0.00
EDDS-Silica	21.77	50.67	25.02	2.54	0.00
EDDS-Co^2+^-Silica	22.85	49.69	24.91	2.22	0.33

**图3:**
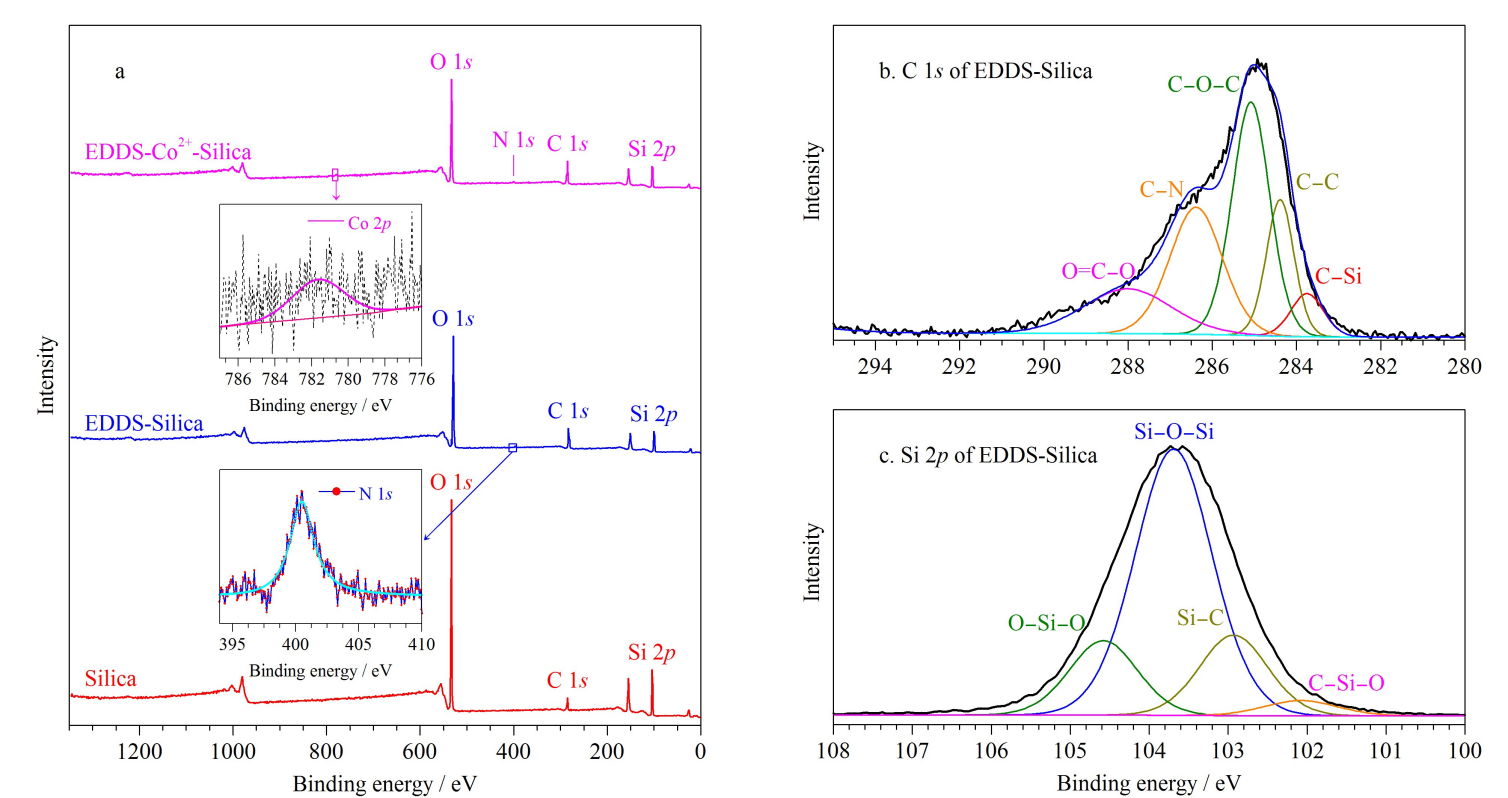
改性材料的X射线光电子能谱图

分别对两种改性材料的键合量进行测定(详见附图1和附表1, https://www.chrom-China.com)。当硅胶与EDDS的质量分别为1.5 g和6.0 g时,EDDS在硅胶基质上的键合量高达170.03 μmol/g。ICP-AES测试结果显示,Co^2+^在EDDS-Co^2+^-Silica材料上的键合量为1.06 mg/g。

### 2.2 金属Co^2+^与EDDS的DFT计算

前期的定性及定量表征只是说明成功改性,但金属Co^2+^与EDDS-Silica之间的作用方式还需进一步确定。以金属Co^2+^与EDDS-Silica作用前后的结合能为优化依据,对其进行模拟和量子计算。结构经优化后,金属离子与配体之间的6个配位键长均发生了改变(见附表2),根据式2计算出优化前后能量变化为-3.12 eV,最终得到EDDS-Co^2+^的稳定配位结构(图4)。由优化结构可知,1分子的Co^2+^与2分子的EDDS可通过配体上的羰基氧、羟基氧及双侧亚氨基氮形成稳定的金属螯合材料。

**图4:**
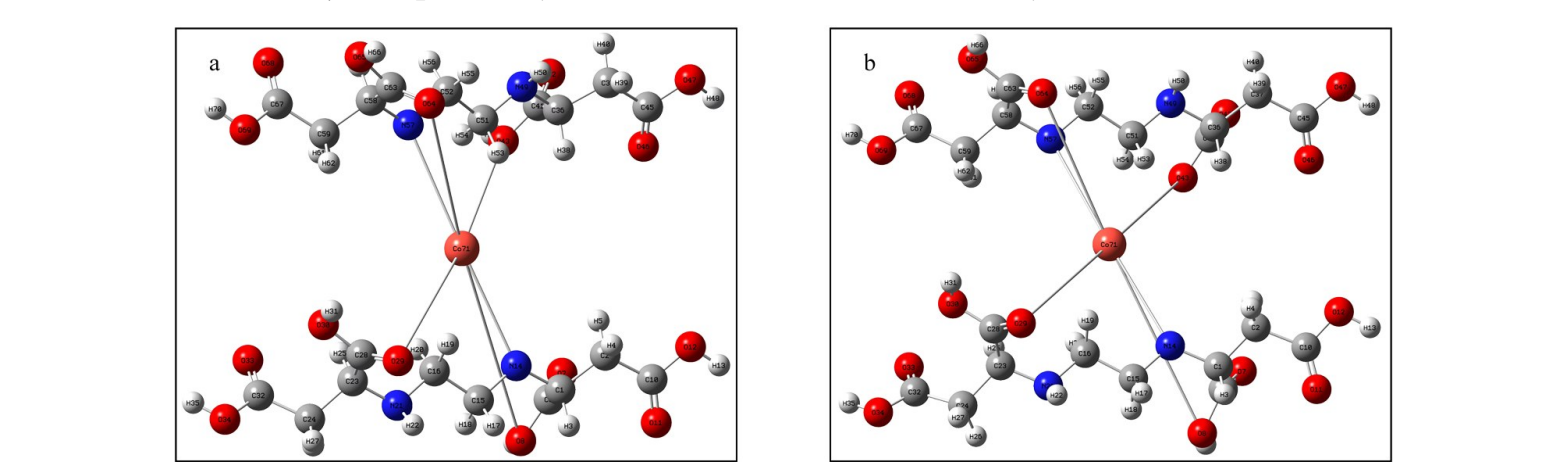
优化(a)前、(b)后EDDS-Co^2+^-Silica的结构图

### 2.3 改性材料对MG一元体系的降解

为克服传统Fenton反应仅适用于酸性废液(pH<3)的局限,我们在Fenton反应中引入不同形式的固体改性材料EDDS-Silica和EDDS-Co^2+^-Silica,在非均相Fenton体系中考察降解MG的适宜pH范围,结果如图5a所示。

**图5:**
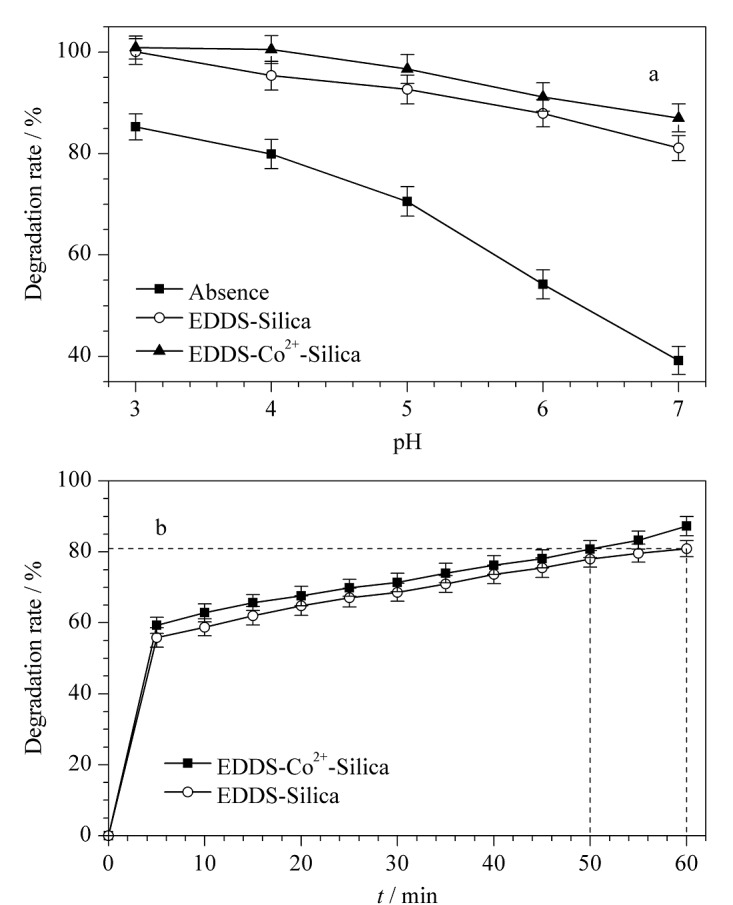
改性材料的催化性能测试(*n*=3)

仅用Fenton试剂时,在pH 3的条件下,MG的最佳降解率为85.27%;随着pH升高,MG的降解率呈明显的下降趋势,pH 7时仅为39.18%。这是因为Fenton试剂的氧化产物Fe^3+^,在pH较高的条件下会形成大量的Fe(OH)_3_沉淀,从而阻碍催化反应的循环进行^[28]^。

在上述pH范围内,两种改性材料加入均可将MG降解率提升至80.88%以上,说明添加EDDS-Silica或EDDS-Co^2+^-Silica均可对MG的降解产生显著改善,这主要表现在3个方面:(1)固体改性材料的添加,使得原有的均相Fenton反应体系变为非均相;(2)在非均相Fenton体系中,EDDS可与金属铁离子形成稳定的配合物,阻止Fe(OH)_3_的形成,因此pH的升高对MG的降解影响不大;(3)在改进的体系中,MG的降解明显优于传统的均相体系,且由图5b可知,EDDS-Co^2+^-Silica对MG的降解率及降解速率均优于EDDS-Silica。在达到相同降解率时,降解时间可由60 min缩短至50 min。


① Fe^2+^+H_2_O_2_→ Fe^3+^+·OH+OH^-^

② Co^2+^+H_2_O_2_→ Co^3+^+·OH+OH^-^

③ Fe^3+^+H_2_O_2_→ Fe^2+^+HO_2_·+H^+^

④ Co^3+^+H_2_O_2_→ Co^2+^+HO_2_·+H^+^

⑤ H_2_O_2_+·OH→ HO_2_·+H_2_O

由式①~⑤Fenton反应的机理可知,在MG的降解过程中,两组氧化还原对Fe^2+^/Fe^3+^、Co^2+^/Co^3+^协同循环促进类Fenton反应的进行,催化H_2_O_2_产生大量的·OH以及HO_2_·^[29]^。MG结构中的显色基团C=C或C=N最先受到·OH/HO_2_·攻击发生断裂,致使MG脱色降解^[30]^。

通过DFT计算,进一步明晰地论证了活性氧对MG分子的攻击位点(见图6)。图中给出了MG的分子结构,用最低未占用分子轨道(LUMO)和最高占用分子轨道(HOMO)描述MG在降解反应过程中容易捕获电子或逸出电子的位点。结果显示,LUMO电位为0.0035 eV,离域电子分布在C1-C6、C15-C19、C25-C29原子上,说明这些原子容易接受电子,容易受到亲核自由基的攻击。与此同时,HOMO电位为-0.1758 eV,离域电子主要位于上侧两个苯环,C14、C21、C24、C29、C31及N34、N43原子容易失去电子受到亲电物种·OH的攻击。

**图6:**
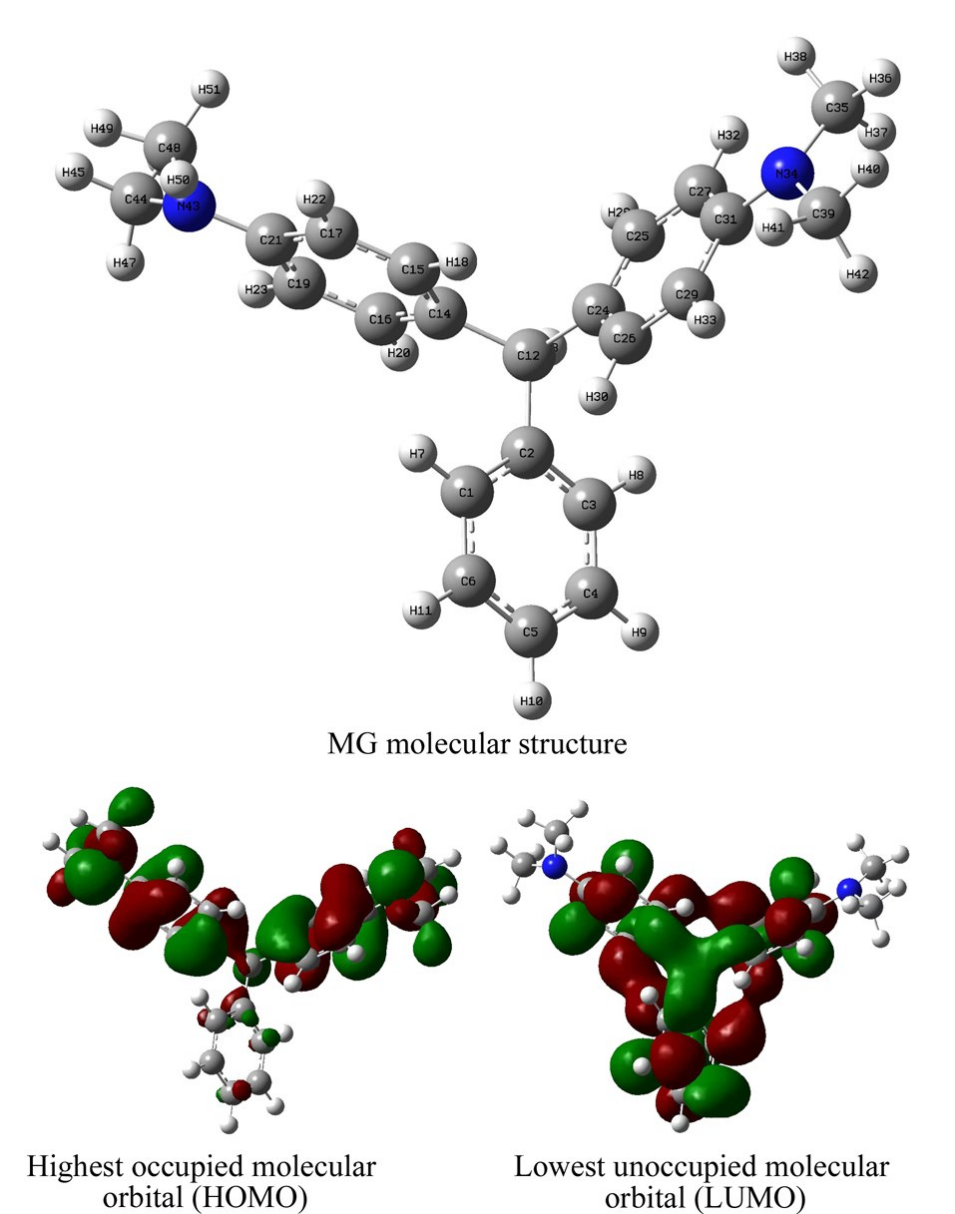
MG分子结构及分子轨道模型

上述机理分析表明,利用EDDS的强螯合性与金属离子形成了稳定配体,在整个类Fenton体系中,Fe^2+^与Fe^3+^及Co^2+^与Co^3+^之间的氧化还原循环催化H_2_O_2_产生更多的·OH,进一步提高了MG降解效果和效率。由此可见,EDDS-Silica、EDDS-Co^2+^-Silica对Fenton反应均具有催化性能。

### 2.4 改性材料对MG及Cr(Ⅵ)二元体系的协同去除及最佳条件探索

#### 2.4.1 pH的影响

如图7a所示,在pH为3~7范围内,MG降解率超过96.67%,Cr(Ⅵ)去除率达94.88%以上,随着pH升高,Fe^3+^水解产生氢氧化物沉淀,导致MG降解率及Cr(Ⅵ)去除率降低。然而,由于EDDS-Co^2+^-Silica改性材料的存在有效扩大了传统Fenton反应的pH应用范围,MG降解率及Cr(Ⅵ)去除率均只有小幅度下降。在此pH范围内,EDDS-Silica改性材料对总Cr及总Fe去除效果显著,总Cr去除率可达80.15%以上,残余质量浓度不超过4.96 mg/L;残余总Fe的含量随pH的增加而降低,且残余质量浓度不超过1.52 mg/L。以上研究结果证明,在对MG和Cr(Ⅵ)二元体系进行协同去除时,EDDS-Co^2+^-Silica的加入可拓宽传统Fenton反应的pH应用范围至中性状态下。因此后续实验均在pH为7下进行。

**图7:**
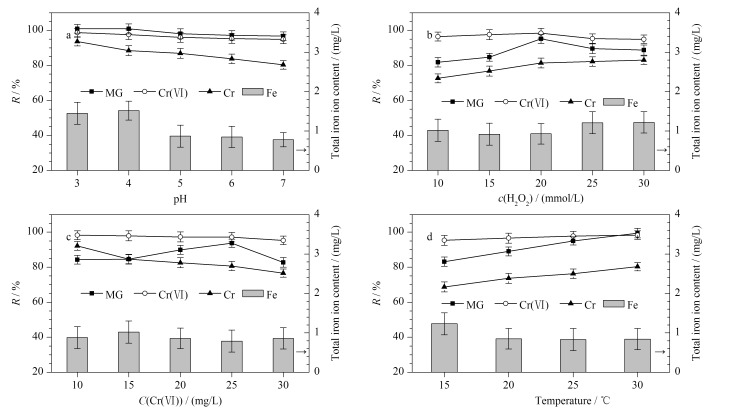
(a)溶液pH、(b) H_2_O_2_初始浓度、(c) Cr(Ⅵ)初始含量和(d)反应温度对MG降解率及重金属离子去除率的影响(*n*=3)

由图5a和图7a对比结果可知,在pH 3~7范围内,二元体系中MG的降解率明显高于一元体系。特别是在pH=7时,二元体系中MG降解率在1 h内可提高至96.67%,相较于一元体系提高了约10%。这一结果一方面再次证实了EDDS-Co^2+^-Silica的加入有利于MG及Cr(Ⅵ)的协同去除;同时也证实了MG与Cr(Ⅵ)之间存在正向协同作用,这主要是因为H_2_O_2_还原Cr(Ⅵ)生成了高反应性的·OH,且材料上的还原性物质(Fe^2+^、Co^2+^)与强氧化性的Cr(Ⅵ)发生反应,在一定程度上减少了对·OH的消耗,提高了对MG的降解效率。在氧化降解MG的同时,实现了将高毒Cr(Ⅵ)绝大部分还原为低毒Cr(Ⅲ)的目标。

#### 2.4.2 H_2_O_2_浓度的影响

在Fenton反应中H_2_O_2_添加量直接决定着反应过程中·OH的生成量。H_2_O_2_添加量的影响如图7b所示,当H_2_O_2_浓度在10~20 mmol/L范围内时,随着添加量的增加,·OH的生成量逐渐增大,促进了MG的降解,与此同时,Cr(Ⅵ)还原为Cr(Ⅲ)的效率也有所提升,使Cr(Ⅵ)去除率增大。在pH为7时,带负电的改性材料通过强螯合、弱静电作用的协同作用更容易吸附Cr(Ⅲ),总Cr去除率增加,最低残余质量浓度为4.26 mg/L;残余总Fe的含量随H_2_O_2_浓度的升高略有增加,但残余质量浓度未超过1.22 mg/L。当H_2_O_2_浓度超过20 mmol/L时,MG降解率存在明显的下降趋势,由2.3节中式⑤可知,过量的H_2_O_2_会与·OH结合,可能会导致H_2_O_2_和·OH的无效消耗,导致MG降解反应效率降低^[31]^。因此,后续实验选择在H_2_O_2_浓度为20 mmol/L下进行。

#### 2.4.3 Cr(Ⅵ)含量的影响

由图7c可以观察到,随Cr(Ⅵ)初始含量的增加,MG降解率呈现先增加后下降的趋势。由于强氧化性的Cr(Ⅵ)被还原,促进类Fenton反应,产生更多的·OH;但当含量增加到一定程度时,部分低价态的金属Cr(比如Cr(Ⅴ)、Cr(Ⅲ))也会被·OH重新氧化,造成·OH的无效使用,从而不利于二者之间的正向协同作用^[32]^。Cr(Ⅵ)主要以HCr $O_{4}^{-}$ 、Cr $O_{4}^{2-}$ 等铬盐形式存在,其被还原为Cr(Ⅲ)的能力有限。相比之下,带负电的EDDS-Silica改性材料通过强螯合、弱静电作用更容易吸附Cr(Ⅲ),导致总Cr去除率出现下降趋势;残余总Fe的含量随Cr(Ⅵ)初始浓度的变化不显著,残余质量浓度约为1.02 mg/L。因此后续实验均在Cr(Ⅵ)质量浓度为25 mg/L下进行。

#### 2.4.4 温度的影响

考虑到MG降解可能是一个吸热过程,实验进而探究了不同温度下EDDS-Co^2+^-Silica对MG-Cr(Ⅵ)双组分废水模拟液的去除效果,结果如图7d所示。温度的上升伴随着MG降解率及Cr(Ⅵ)去除率的提高。可能是由于温度升高促进了·OH的生成,有助于MG的快速降解;且高温有利Cr(Ⅵ)的还原,Cr(Ⅵ)的去除率也有所增加。总Cr的去除率随着温度的升高而增加,可能是因为高温会影响扩散速率,从而有利于Cr(Ⅵ)的还原以及改性材料对总Cr的吸附;残余总Fe的含量随温度的升高而下降,且最大残余质量浓度不超过1.23 mg/L。因此,温度为30 ℃时可达到最佳去除效果。

根据国家及各地方的环保规定,全国一般工业及其他污染源排放的总Cr质量浓度规定为不得超过50 mg/L,主要河流及重点水域排放的质量浓度不得超过5 mg/L; 《污水排入城市下水道水质标准》等总Fe含量排放标准规定的质量浓度不得超过5 mg/L。综上,本研究所用EDDS-Silica处理双组分废水模拟液总Cr及总Fe的残余量均符合国家排放标准。

## 3 结论

本研究提出的氨基多羧酸类复合金属改性材料作为非均相类Fenton催化剂,具有催化Fenton反应和吸附去除残余金属离子的双重性能,在处理染料降解及重金属离子废水方面具有广阔的应用前景,同时也为其他同类新型配体改性材料的开发提供了参考价值。
